# Reply to Frański, R.; Beszterda-Buszczak, M. Comment on “Villalva et al. Antioxidant, Anti-Inflammatory, and Antibacterial Properties of an *Achillea millefolium* L. Extract and Its Fractions Obtained by Supercritical Anti-Solvent Fractionation against *Helicobacter pylori*. *Antioxidants* 2022, *11*, 1849”

**DOI:** 10.3390/antiox12071384

**Published:** 2023-07-04

**Authors:** Marisol Villalva, Jose Manuel Silvan, Teresa Alarcón-Cavero, David Villanueva-Bermejo, Laura Jaime, Susana Santoyo, Adolfo J. Martinez-Rodriguez

**Affiliations:** 1 Microbiology and Food Biocatalysis Group (MICROBIO), Department of Biotechnology and Food Microbiology, Institute of Food Science Research (CIAL, CSIC-UAM), C/ Nicolás Cabrera, 9, Cantoblanco Campus, Universidad Autónoma de Madrid, 28049 Madrid, Spain; 2 Microbiology Department, Hospital Universitario de La Princesa, 28006 Madrid, Spain; 3 Department of Preventive Medicine, Public Health and Microbiology, School of Medicine, Autonomous University of Madrid, 28029 Madrid, Spain; 4 Department of Production and Characterization of Novel Foods, Institute of Food Science Research (CIAL, CSIC-UAM), C/ Nicolas Cabrera 9, Cantoblanco Campus, Universidad Autónoma de Madrid, 28049 Madrid, Spain

Franski and Beszterda-Buszczak [1] report some errors made in the identification of compounds in *Achillea millefolium* extract by MS/MS analysis included in the supplementary material of our published article [2]. We thank them for the observations, and we are pleased to be able to clarify the doubts from these authors. The following response offers an analysis of the comments made, compound by compound.

Regarding apigenin identification, as shown in Figure 1, spectrum 269 remains as the main product after MS/MS analysis along with other product ions such as 151 or 117, which have been reported as characteristics of apigenin fragmentation elsewhere [3,4,5,6]. In addition, *m/z* at 112.9858 was found for apigenin after MS/MS analysis. According to this finding, it was the only fragment included in Table S1, although 151 and 117 could also be included. 113 ion is in accordance with Taamalli et al. [3], who found it to be one of the product ions for apigenin-*O*-glucuronide. Therefore, a typographical error would be attributable, in this case, by reflecting 112 instead of 113. Moreover, based on its accurate mass, C_15_H_10_O_5_ (corresponding to the molecular formula of apigenin) was proposed for this product with an error of 3.2 ppm. In addition, the UV-Vis spectrum (data not included in this manuscript) and retention time matched those corresponding to the authentic apigenin standard.

As can be seen in Figure 2, diosmetin yielded the ions 299 (100), 284 (55), and 256 (12) as the main ion products, corresponding to a characteristic fragmentation ion from diosmetin [7]. No other ions were found in this analysis (e.g., 227, 151, or 107). However, based on the accurate mass [M−H]^−^ at 299.0554, the molecular formula C_16_H_12_O_6_ (error 1.2 ppm) was obtained, which corresponded to diosmetin. Moreover, further identification was performed according to the UV-Vis spectrum and the retention time compared to diosmetin’s authentic standard. Therefore, 112 ion, currently registered in Table S1, is a typographical error, and the omitted product ions (284 and 256) should be included in Table S1.

Franski and Beszterda-Buszczak noted an incorrect assignment of [M−H]^−^ at 315 as methoxyquercetin isomer. In the present study, the molecular formula C_16_H_12_O_7_ resulted with the *m/z* of [M−H]^−^ at 315.0508 with an error of −3.3 ppm. The fragmentation pattern resulted in 300 ion (100) as the main product. Accordingly, a loss of CH_3_ (15 Da) could suggest the loss of [M−H]^2−^ instead of 14 Da, and therefore the loss of [M−H], corresponding to 301 as the fragmentation ion. Franksi and Beszterda-Buszczak also suggest the possibility of examining other product ions, such as isorhamnetin glycoside, which was reported by Dias et al. [8] in *Achillea millefolium* L. However, the mass [M−H]^−^ at 477 was not detected in our case. Certainly, other product ions were considered for preliminary analysis. In this regard, we considered the authentic standard of isorhamnetin, which corresponds to 3′-*O*-methylquercetin (also known as 3′-methoxyquercetin in the literature), but its retention time did not correspond with any of the identified compounds for *Achillea millefolium* L. Therefore, we have used methoxyquercetin, as its generic name, instead of *O*-methylquercetin.

Regarding amentoflavone identification, there was an error about the fragmentation ions of this compound shown in the HPLC-MS/MS spectra in Table S1. As can be seen from the fragmentation pattern shown in Figure 3, the characteristic amentoflavone product ions were detected but not reported properly according to the *m/z* at 375 (100), 443 (10), and 417 (20). This is in accordance with the literature for amentoflavone product ion mass spectra [9,10]. In addition, amentoflavone was designated by comparing its UV-Vis spectra and retention time using an authentic standard. Hence, the fragmentation pattern for amentoflavone in Table S1 should be modified.

Franski and Beszterda-Buszczak also mentioned the product ions of three isomers of flavone *C*-glycosides: apigenin-*C*-hexoside-*C*-pentoside, schaftoside, and schaftoside isomer. They claimed that the product ions—and their abundance—are similar for the three reported compounds. Certainly, no other product ions were detected for these isomers, although the accuracy of the *m/z* product ions varied slightly (with an accuracy within four decimal places), along with their relative abundance. Hence, the relative abundance displayed in Table S1 for apigenin-*C*-hexoside-*C*-pentoside, schaftoside, and schaftoside isomer should be updated.

For luteolin-6,8-di-*C*-glucoside with an [M−H]^−^ ion at *m/z* 609, the molecular formula C_27_H_30_O_16_ was found for the most likely compound with an error of 1.6 ppm. The most reported MS/MS fragmentation pattern included a fragmentation ion of [M−H−120]^−^ from *m/z* 609, corresponding to a neutral loss of sugar residue, which yields the main ion fragment at *m/z* 489 [3]. The second fragment ion yielded a mass (*m/z*) of 325, which Franski and Beszterda-Buszczak did not recognize as a characteristic product ion. Surely, common fragmentation patterns in a negative ion mode include an ion at *m/z* 327 for this compound [11]. We decided to include the questioned ion fragment (*m/z* at 325) since luteolin possesses four -OH radicals, and it is possible that further ionization can occur, resulting in three hydrolyzed -OH radicals by means of a loss of [M−3H]^3−^.

For the product ions of cryptochlorogenic and chlorogenic acid, an inaccuracy exists in the reported fragmentation pattern in Table S1. The correct *m/z* of the main product ions correspond to 191 (100) for chlorogenic acid and 179 (67) and 173 (100) for cryptochlorogenic acid. Therefore, Table S1 should be updated. It is worth mentioning that these three chlorogenic acid isomers were also identified via a comparison with their authentic standards, as was already indicated in Table S1.

The identification of Vicenin 2 product ions was omitted by error but has now been updated and shown in Table S1. As can be observed in Table S1, the [M−H]^−^ at *m/z* 593.1513, its corresponding molecular formula C_27_H_30_O_15_ (with an error of −0.2 ppm), and its main ion product *m/z* at 473 (100) are suggested to be the correct identification of Vicenin 2. In addition, an authentic standard was used to elucidate the proper identification. Vitexin was misclassified in Table S1 since it appeared in the flavonols section. Now, vitexin can be found in the flavones section since it is a flavone glycoside derivative of apigenin. The accurate mass *m/z* of ferulic acid was reported with a typographical error; the correct mass [M−H]^−^ corresponds to *m/z* 193.0504. This mass has been corrected in Table S1.

Thus, after the revisions and modifications reflected in this letter, we would like to state that we have clarified all doubts. In addition, the results already published have full rigor and quality according to the standards of the scientific community and the procedures of the journal itself.

## Figures and Tables

**Figure 1:**
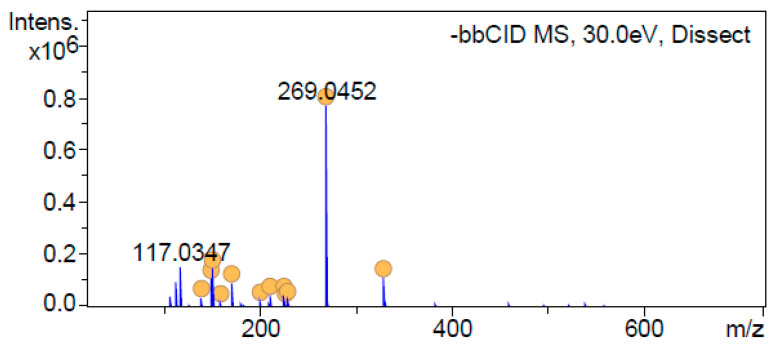
MS/MS spectrum peak identified as apigenin.

**Figure 2:**
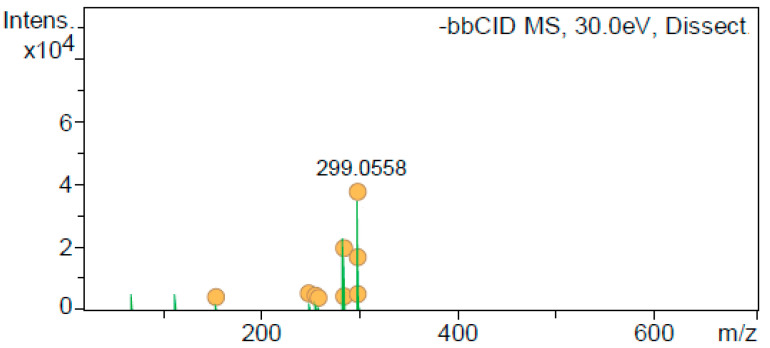
MS/MS spectrum peak identified as diosmetin.

**Figure 3:**
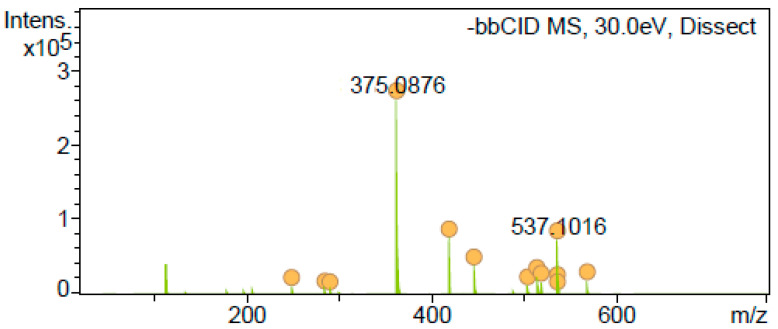
MS/MS spectrum peak identified as amentoflavone.

## Data Availability

The data presented in this study are available in the article and supplementary materials.

## Supplementary Materials

The following are available online at https://www.mdpi.com/article/10.3390/antiox12071384/s1, Table S1: Phenolic compounds identified in yarrow samples by using HPLC-ESI-QTOF-MS.
